# Assessing the accuracy of 3D assistive technologies for surgical guidance of osteosarcoma resections: a comparative laboratory study of mixed reality, patient-specific instruments and freehand approaches

**DOI:** 10.1186/s42836-026-00369-8

**Published:** 2026-02-05

**Authors:** Jose Caceres-Alban, Dieter M. Lindskog, Johannes M. Sieberer, Alyssa Glennon, Steven M. Tommasini

**Affiliations:** 1 Yale School of Engineering & Applied Science, New Haven, CT 06511 USA; 2 Yale School of Engineering & Applied Science - Mechanical Engineering, New Haven, CT 06511 USA; 3 Yale School of Medicine - Orthopaedics & Rehabilitation, New Haven, CT 06520 USA

**Keywords:** Mixed Reality (MR), Patient-specific Instrumentation (PSI), Osteosarcoma, Computer-Assisted interventions, 3D-printing, Resection Margins

## Abstract

**Background:**

The survival rate after surgical osteosarcoma resection is low, particularly when the sarcoma is not fully removed. Therefore, wide surgical margins are used in surgery, limiting how much bone can be salvaged. Patient-specific instrumentation (PSI) enables smaller margins, but utilization is low. Mixed reality-based techniques (MR) might be easier to implement. The purpose of this study was to compare the cutting accuracy of MR, PSI, and freehand techniques in 3D-printed osteosarcoma models and determine the corresponding technique-related minimal surgical margins.

**Methods:**

CT-scans of patients with extremity osteosarcoma were acquired, segmented, and the bones 3D-printed three times. Scans were excluded if they had low resolution or metal artifacts. Pre-surgical planning for full resection was conducted, and corresponding PSI and MR plans were created. Tumor resections were separately done via a freehand, PSI, and MR approach. Resected bone models were 3D scanned, and the cutting accuracy was determined. Differences in accuracy were determined via Bartlett’s test and corresponding post-hoc tests for a significance level of 0.05. The techniques’ surgical margins were determined for 90, 95, 97.5, and 99% successful cuts.

**Results:**

Ten osteosarcomas with one to three cutting planes were included, leading to a total of 19 cuts. The variance in cut accuracy was significantly greater for the freehand approach (standard deviation (STD) [95%CI]: 6.85 [5.18–10.13] mm) than the MR (STD: 3.71 [2.79–5.57] mm) and the PSI (STD: 2.68 [2.02–3.96] mm) approach. No significant difference in variance between PSI and MR was found (*P* = 0.44). Surgical margins varied between techniques, with the freehand approach being about twice those of the MR and PSI approaches. To achieve 99% tumor-free cuts, the estimated required margins were 15.9 mm (freehand), 6.2 mm (PSI), and 8.6 mm (MR).

**Conclusion:**

This study acts as a non-clinical proof of concept that the adoption of patient-specific instrumentation or mixed reality techniques for osteosarcoma resection might enable narrower margins pending in-vivo validation, potentially enabling bone and joint preservation and restoration, while decreasing resection failure rates.

**Supplementary Information:**

The online version contains supplementary material available at 10.1186/s42836-026-00369-8.

## Introduction

Osteosarcoma is the most common primary bone cancer, accounting for 55% of all cases in patients under 24 years old in the US [1]. Nearly 20% of osteosarcoma cases are metastatic at diagnosis. These tumors frequently originate near the metaphysis of long bones, primarily in the femur (42%), tibia (19%), and humerus (10%) [2].

Osteosarcoma management, like with other malignant bone tumors, is highly dependent on the surgical resection of the tumor zone [3]. While the primary treatment objective is timely oncological resection, the functional goal of limb or joint preservation remains critically important for the patient. This priority, and the increasing use of computed tomography (CT) scans and magnetic resonance imaging (MRI), has promoted the selection of limb-salvage and joint-preservation surgeries over amputations.

It is estimated that 9 out of 10 patients with extremity sarcomas can be treated with limb-salvage surgery [4]. Surgeons should resect the tumoral zone while cutting through the surrounding healthy tissue with sufficient clearance to ensure complete tumor removal. The goal is to preserve as many of the surrounding structures as possible to maintain limb functionality. Still, full resection of the osteosarcoma is essential, as mortality increases by a factor of 5.1 when the cut leaves some of the tumor remaining [4, 5].

This has led to the establishment of surgical margins [6, 7]. Among those, wider margins have demonstrated a positive effect on reducing the risk of local recurrence and metastatic spread while preserving acceptable limb functional outcomes [8], but may result in restricted limb function, limb length discrepancies, and limited reconstructive options [9]. The ideal margin depends on the anatomy, the osteosarcoma’s shape, the deployed technique, and the quality of pre-surgical imaging and planning.

The most widely established resection approach involves freehand cutting guided by a surgical margin calculated from bidirectional images derived from CT scans or MRI. However, this technique is associated with an estimated 55 to 65% risk of failure in both experienced and junior surgeons when attempting to achieve a 5 mm surgical margin [10], suggesting that larger margins may be required. Over the last decade, three-dimensional (3D) assistive technologies integrated into surgical workflows have proven to improve patient outcomes [11]. 3D printed patient-specific instruments (PSI) have shown positive effects as guiding tools for performing osteotomies and reduction of safe surgical margins [12, 13]; however, their applicability in hospital settings remains limited due to associated costs and entry barriers. These include either purchasing from specialized industry firms or creating a dedicated point of care 3D printing center. Such centers require, among others, the integration of high-resolution 3D printers, biocompatible materials, and sterilization processes within the clinical site. Additionally, extended manufacturing timeframes may pose further disadvantages.

In this context, mixed reality (MR) emerges as a novel intraoperative solution. It uses digital holographic projections overlaid on the patient’s anatomy to guide surgical procedures, such as drilling and cutting. This minimizes the need for physical surgical guides and provides real-time feedback on the orientation and position of surgical instruments. To date, this technology has been tested and FDA-cleared for spinal surgery and shoulder arthroplasty, among other intraoperative applications. Despite this, its applicability and level of accuracy in onco-orthopedic osteosarcoma resections remain minimally explored.

The purpose of this study was to compare the cutting accuracy of MR, PSI, and freehand techniques in 3D-printed osteosarcoma models and determine the corresponding technique-related minimal surgical margins.

## Methods

The protocol for this study was approved by our Institutional Review Board (Protocol ID: 2,000,038,760). Patients from our institution’s medical record database primarily diagnosed with osteosarcoma were selected and included if high-resolution medical images were available. Exclusion criteria of the medical images were: (1) presence of metallic implants interfering with segmentation, (2) substantial gaps between slices, and (3) cases with only MRI and no CT imaging. The images were obtained as DICOM-typefiles. Seven osteosarcoma cases were selected, and three additional ones were downloaded from Embodi3D (Bellevue, WA, USA) according to the same criteria, including anatomical structures such as the humerus, tibia, and femur.

### Virtual surgical planning and printing of 3D models

Segmentation and virtual surgical planning were performed for all cases. A biomedical engineer, supervised by an orthopedic surgeon, segmented and created 3D models of the full bone and the tumor, using Materialise Mimics v26.0 software (Materialise MV, Leuven, Belgium). The orthopedic surgeon determined the surgical approach, the orientation of the resection planes, and the safety margins on each case. Each full bone model was 3D printed three times with Grey Resin v4 on a Form 3BL 3D printer (Formlabs, Somerville, MA, USA) and allocated into three groups: freehand, PSI, and MR.

Additionally, patient-specific instruments (surgical guides) were designed to guide the planned osteotomies, using 3-matic 18.0 software (Mimics Innovation Suite, Materialise MV) and were created per the surgeons’ specifications about the safety margins and osteotomy planes. The surgeon was a practicing fellowship-trained orthopaedic surgeon from our department. The guides included non-parallel hollowed cylinders added to the surgical guide to insert 1.2 diameter Kirschner wires and allow their proper fixation on the bone models. The guides were printed using the same material and printers as the bone models. The same surgical cuts were used for the MR approach.

### Surgical resection

**Fig. 1:**
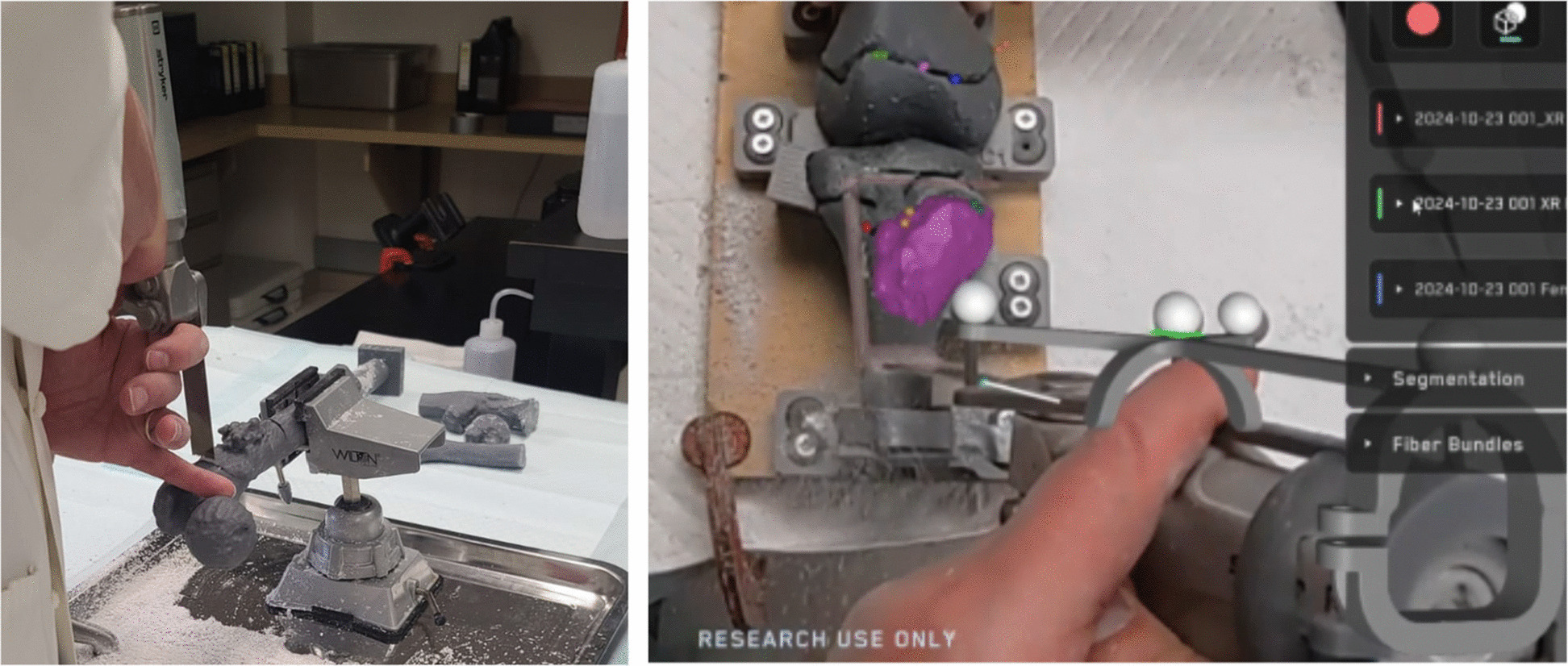
Freehand cut procedure (left) and surgeon’s view using the MR approach (MEDIVIS, SurgicalAR) for guiding the resection osteotomy (right)

The physician performed each resection using three different techniques: traditional freehand, PSI, and MR. The freehand approach was done without the aid of additional surgical guidance, based solely on the preoperatively defined plan. A ruler and a marker were provided to the surgeon to manually transfer the planned measurements onto the 3D-printed anatomical model. Then the surgeon used the 3D-printed surgical guides for the resection by applying them and performing the cuts. Lastly, the mixed reality cuts were performed utilizing SurgicalAR® research MR software from Medivis Inc (New York, NY, USA) and a second-generation HoloLens® (HL2) by Microsoft Corporation (Redmond, Washington, USA) (see Fig. 1). The surgeon completed two 20-min training sessions with all three techniques before the actual experiment.

The required time was recorded for each bone model. The clock started for the freehand method upon marking of the bone, for PSI as soon as the guide touched the bone, and for MR when the CT-scan was registered on the bone model. The clock was stopped after the osteotomy was finished. Marking the bone, placing the guide, and registering the bone were done by the surgeon.

### Surgical cut accuracy analysis

Postoperative 3D scans of each 3D-printed osteosarcoma model were acquired using the CR-Scan Lizard 3D scanner (Creality, Shenzhen, China) with a resolution of 0.1 mm and an accuracy up to 0.05 mm. A surface registration of the pre- and post-bone models was performed with an iterative closest point (ICP) algorithm in 3-matic 18.0 (Mimics Innovation Suite, Materialise MV).

To assess the volumetric fidelity between the pre- and post-anatomies, the Dice Similarity Coefficient (DSC) was used in each model as a quantitative quality control metric[14]. The DSC was calculated using Mimics v26.0 software (Mimics Innovation Suite, Materialise MV) and two segmentation masks: test and reference. The test mask was derived from the cut bone segment with the largest surface area in each case, while the reference mask was obtained from the Boolean intersection of the original (uncut) bone and the test mask.

The accuracy of each cut was assessed by digitally measuring the greatest distance (in mm) between the intended and the current osteotomy plane cuts. This process is visualized in Supplemental Figs. S1 and S2. This parameter was described by *Cartiaux* et al. as Location Accuracy (LA), and it’s based on their ISO-based evaluation for the accuracy of computer-aided techniques in orthopedic surgery [10]. A bone loss of 1.5 times the saw blade thickness was considered the kerf. A half-kerf correction was applied to the LA measurement: half a kerf was added for over-deviated cuts and subtracted for under-deviated cuts [15].

### Data analysis

Cut deviations for each group were assessed for normality using the Shapiro–Wilk test. Values above the significance level indicate normality. A Kruskal–Wallis Test was employed to compare differences in cut means. Bartlett’s test was employed to compare variance across surgical techniques. Post hoc pairwise comparisons were conducted using the F-test. Surgical margins for 90.0, 95.0, 97.5, and 99.0% successful cuts and their confidence intervals were calculated based on the assumption of normality. Cutting time differences were evaluated using the Kruskal–Wallis test. A significance level of *P* < 0.05 adjusted via Holm-Bonferroni was used for all tests.

## Results

**Fig. 2:**
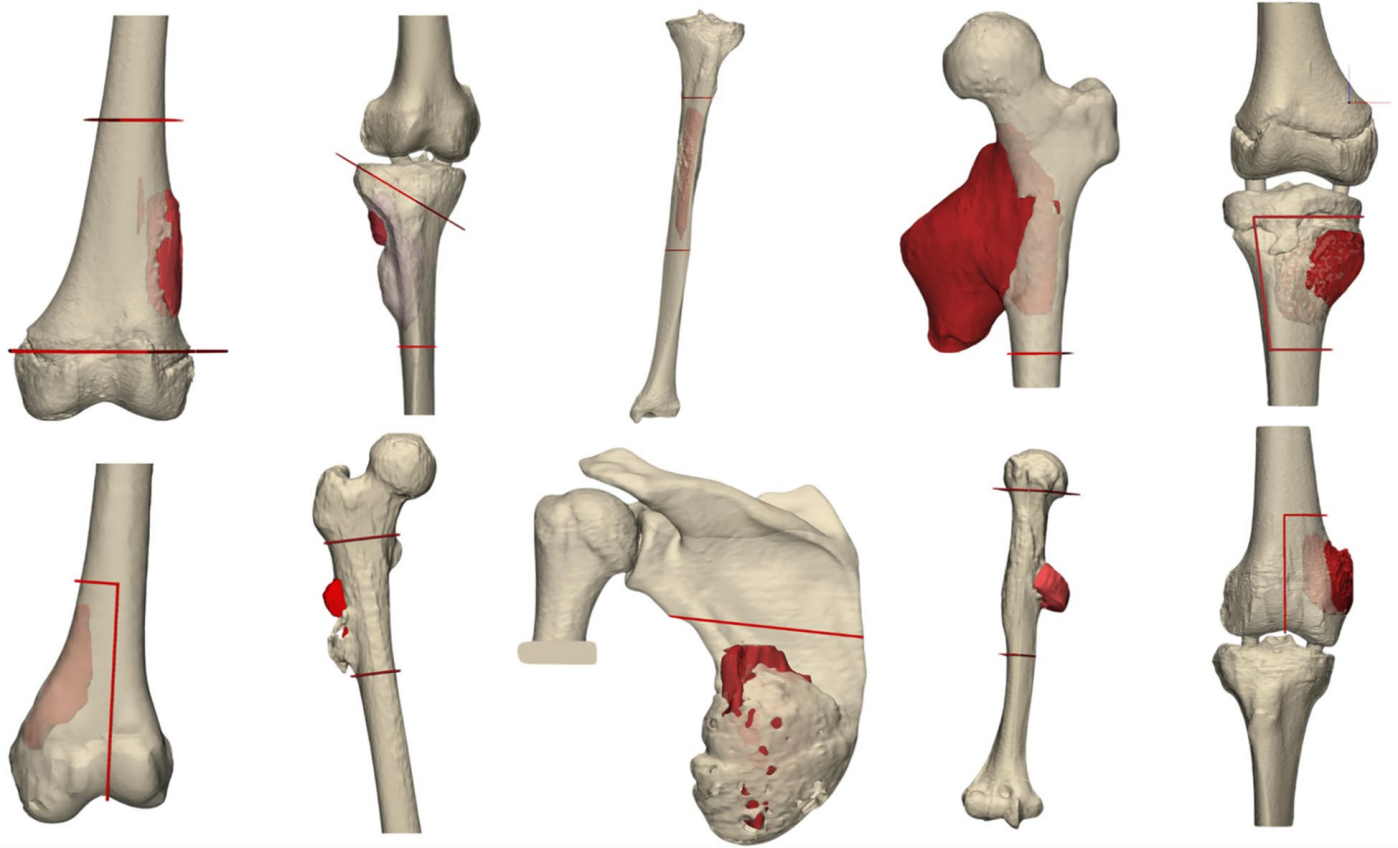
Segmentations of osteosarcoma cases and cutting trajectories defined by the surgeon after the virtual surgical planning session. Bone is represented in pearl color, tumor in red wine, and cutting planes in red. Three resections were done with a 5 mm margin to avoid cutting too much into articulate surface and enable salvaging of the respective joint

Seven patient scans from our institution’s database, and three from the public database, were included, resulting in a total of 10 patients with 19 cut trajectories. Resection safety margins were set at 10 mm for 16 cuts and 5 mm for the other three (see Fig. 2).

The resections were performed for each group by the same surgeon, and post-resection 3D scans were conducted. In total, 56 cuts were performed to evaluate cutting performance using the three technologies: 19 Freehand, 19 PSI, and 18 MR-guided resections. Although 19 resections were initially planned for the MR-guided group, one was excluded because the printed bone model became unstable after the second cut, misaligning the MR system and preventing completion of the procedure. This happened because the long bone was only fixed at its proximal and distal ends, with no support structure under the bone.

The similarity analysis showed a high degree of agreement, with DSC of 0.99275, 0.9908, and 0.99164 for the FH, PSI, and MR methods, respectively, indicating sufficient accuracy of the post-resection scans.

### Location accuracy

**Fig. 3:**
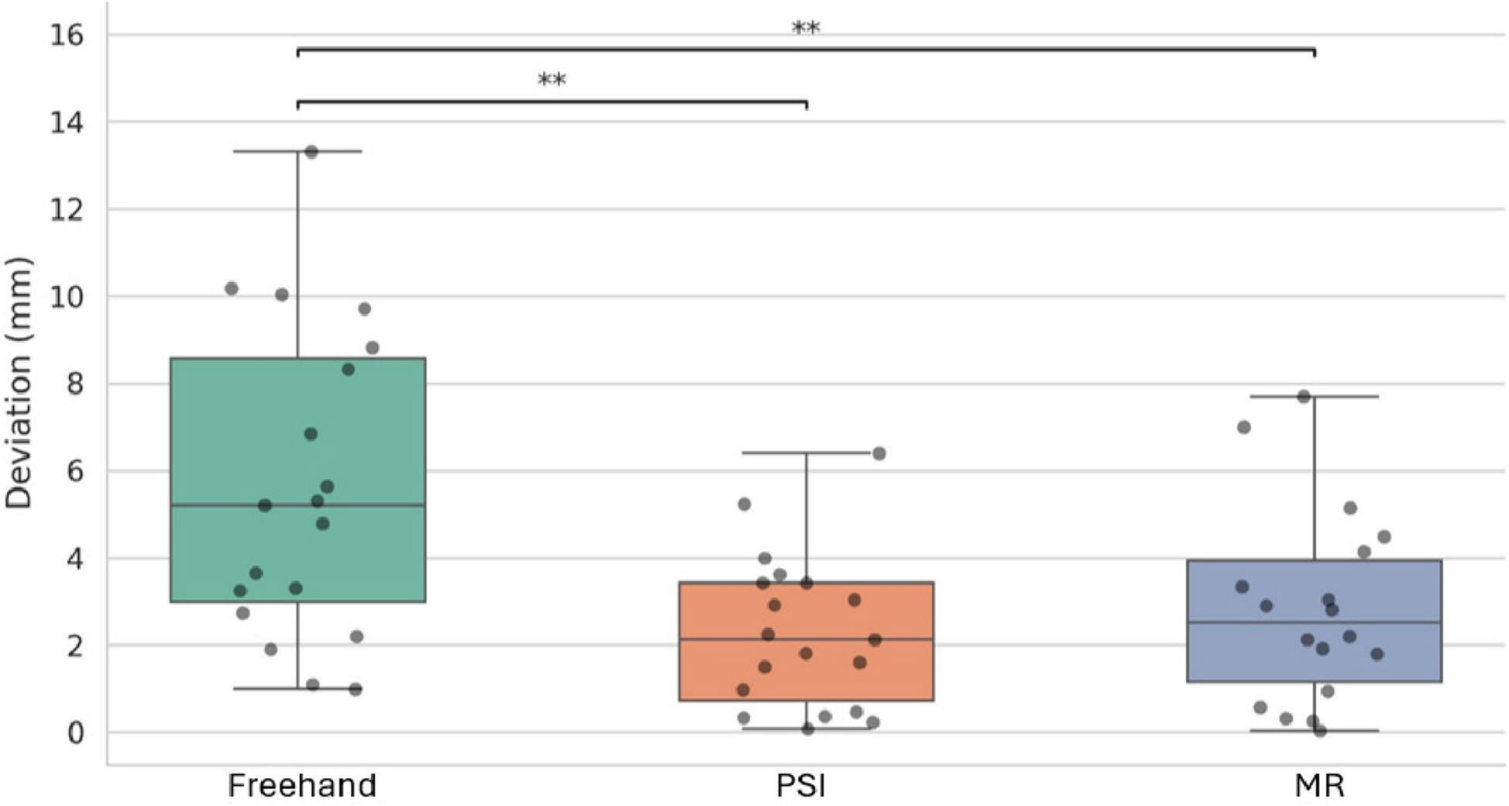
Comparison of location accuracy (in millimeters) by cutting approach (Freehand, PSI, MR). Horizontal lines and asterisks indicate statistically significant differences

The Shapiro–Wilk test indicated that cut deviations were likely normally distributed (*P* = 0.26, 0.23, 0.18). The results of the performed accuracy analysis yielded mean absolute deviations from the ideal cutting plane for freehand (5.65 mm), MR (2.89 mm), and PSI (2.30 mm). The cuts were on average − 0.32 ± 6.85 off for freehand, 0.14 ± 2.68 for PSI, and 1.22 ± 3.71 for MR, with negative values indicating cuts closer to the tumor, measured relative to the ideal cutting plane. Variance (technique accuracy) was significantly lower for the freehand approach compared to PSI (*P* = 0.002) and MR (*P* = 0.015). No significant difference in means (*P* = 0.35) was found. Among the 30 simulated osteosarcoma resections, 29 were completed with margins outside the tumor boundary. Of these, three—each performed using the freehand technique—had resection planes positioned less than 2 mm from the tumor surface. The remaining resection, also performed freehand, resulted in an intralesional cut with a deviation of − 10.04 mm from the planned resection plane. No cases of intralesional cuts or margins below 2 mm were observed in the PSI or MR groups. Detailed results are outlined in Table 1 and visualized in Fig. 3.

**Table 1:** Descriptive statistics of the three resection techniques

Method	MAD	Mean ± SD
Freehand	5.65 mm	− 0.32 ± 6.85* mm
PSI	2.30 mm	0.14 ± 2.68 mm
Mixed Reality	2.89 mm	1.22 ± 3.71 mm

MAD: mean absolute deviation; SD: standard deviation, PSI: Patient specific instrumentation; *denotes statistical difference to other two groups (PSI and Mixed Reality)

The standard deviations of the freehand approach (standard deviation (STD) [95%CI]: 6.85 [5.18–10.13] mm), the MR approach (STD: 3.71 [2.79–5.57] mm), and the PSI approach (STD: 2.68 [2.02–3.96] mm) were calculated. These were used to determine surgical margins for different expected success rates for full resections. The freehand method required significantly higher surgical margins (see Table 2).

**Table 2:** Surgical technique margins for different success rates. None overlapping confidence intervals indicated statistical significance

	**Surgical Margin in mm per Success Rate [95%CI]**			
Method	**90%**	**95%**	**97.5%**	**99%**
Freehand	8.78 [6.64–12.98]	11.27 [8.52–16.66]	13.42 [10.15–19.85]	15.93 [12.05–23.57]
PSI	3.43 [2.59–5.07]	4.41 [3.32–6.51]	5.25 [3.96–7.76]	6.23 [4.70–9.21]
Mixed Reality	4.75 [3.58–7.14]	6.10 [4.59–9.16]	7.27 [5.47–10.92]	8.63 [6.49–12.96]

PSI: Patient specific instrumentation, CI: Confidence interval

Lastly, the operative time required for performing the cutting of the models was not significantly different (*P* = 0.086) between freehand (213 ± 140 s), PSI (250 ± 95 s), and MR (324 ± 136). The raw time data have been plotted in Supplemental Fig. S3.

## Discussion

When comparing resections performed via freehand, patient-specific instrumentation (PSI), and mixed reality guidance (MR) approaches, we found: (1) better accuracy when using PSI and MR, (2) no significant differences between PSI and MR, and (3) a possible reduction of surgical margins when employing these newer techniques.

Studies consistently show that assistive modalities such as AR/MR, computer navigation, and PSI significantly improve osteotomy accuracy compared with conventional freehand techniques. For example, in a controlled pig-femur model, AR-guided cuts demonstrated a mean deviation of approximately 1.7 mm from the planned plane, compared with 2.6 mm using a manual freehand approach [16]. Similarly, a human cadaveric comparison reported mean cutting errors of 1.9 ± 1.1 mm with PSI and 3.6 ± 2.1 mm with computer-assisted navigation, in contrast to 9.2 ± 3.3 mm for conventional freehand osteotomies [15]. The differences observed between these resections highlight the value of 3D-technologies in tumor resection surgery. By employing these tools, surgeons can achieve greater precision and define narrower surgical margins or increase success rates. This could help preserve the limb or joint while minimizing the risk of failed resections. Our accuracy results align with previously reported literature on the advantages of PSI over freehand techniques for tumor resections [13, 15].

The normal distribution of our study results allowed us to define surgical margins leading to 95% confidence that resections will be outside the tumor. PSI and MR showed reduced margins of 4.4 mm and 6.1 mm, respectively, compared to the freehand’s 11.3 mm. This is in line with previous surgical navigational studies like Sternheim et al.’s [17] landmark paper, which was tested on cadavers under simulated surgical conditions. They found that a margin of 5 mm leads to more than 95% cuts being outside the tumor. Furthermore, it is in line with the numbers cited by Bruschi’s 2023 review on surgical navigation techniques [18]. Although our study did not include conventional navigation, the safety margin required for MR guidance was similar to values reported for navigated cuts, performed comparably to PSI, and markedly better than freehand. This suggests that MR may offer a practical and lower-cost alternative to PSI while still providing a meaningful improvement in resection accuracy, albeit with a potential loss of precision.

Mixed reality has been less explored as a surgical navigation tool. Previously, the location accuracy of the system we used has been demonstrated in studies following the FDA-recognized standard ASTM F2554-18 for assessing the location accuracy of computer-assisted surgical systems [19]. Their mean positional tracking error reported was 0.75 ± 0.37 mm. Contrarily, our resection mean absolute deviation was higher with 2.89 mm. While the previous study primarily assessed the precision of the marking system without cutting, ours included the resection. This likely shows that even if the MR markings are accurate, the cut itself will introduce additional deviation. We are assuming MR-based guidance may differ upon use case or could improve with increased operator experience. Still, it seems unlikely that MR-based approaches with experienced operators can achieve substantially higher accuracy than PSI. Furthermore, in a real surgical setting, the deviation introduced by the cut could be further influenced by the presence of soft tissue and vascular structures that may interfere with visibility and the blade.

Evidence from spine, neurosurgery, and craniomaxillofacial (CMF) surgery also shows that AR/MR, navigation, and PSI can improve accuracy and workflow compared with freehand techniques. In CMF oncology, recent work integrating AR with surgical navigation for oral cancer resections reported mean osteotomy deviations of 1.68 ± 0.92 mm and 100% negative bony margins, with no early local recurrences [20]. In spine surgery, Elmi-Terander et al. [21]compared AR-based navigation with freehand pedicle screw placement and found 94% perfect screw placement with AR versus 90% with freehand (*P* < 0.05). These small but significant improvements are consistent with what we might expect in orthopedic oncology, where guided techniques can outperform freehand methods, although often by a modest margin when procedures are performed by experienced surgeons. In contrast, prior studies indicate that AR and navigation confer much larger benefits for less-experienced surgeons, effectively reducing the experience-related performance gap and supporting their use as training and equalizing tools. [16, 22].

One MR model became unstable after the bone was cut twice, making aligning the MR system difficult. This highlights the need for surgeons to be proficient in the freehand technique before adopting supportive technologies. Technology could always fail, potentially preventing completion of the procedure. Lastly, it is unlikely that our test setup will give a good indication of changes in total operating times, given the influence of multiple additional factors. Still, it doesn’t seem like substantial differences between techniques are to be expected. Resections with supportive technology (i.e., MR and PSI) seem to be slower, particularly for MR.

### Limitations

In this study, only one surgeon was included, limiting the generalizability of the results. Furthermore, this surgeon had limited experience with MR and PSI, while primarily employing the freehand method. With respect to the results, we deem this conservative, as accuracy should improve with more experience. Furthermore, as MRI and PSI are new technologies, most adopters will be well versed in the freehand technique while having limited experience in MR or PSI. Having participants with different surgical backgrounds could further change the results. For example, a surgeon with greater experience using PSI and MR would likely achieve higher cut accuracy than the surgeon in this study.

The bone models used were printed with material not equal to bone, changing the cutting performance. Additionally, soft tissues (e.g., muscles and vasculature) were not replicated in the resected bone models, which may affect the accuracy and feasibility of the PSI and MR approaches in real surgical settings. For example, placement of the PSI could be more difficult, or bleeding could impact the visual guidance of the MR system. We also decided not to include placement of the PSI, marking the bone, and registering the bone to the MR system in our time measurement, as the needed time is likely dependent on the surrounding soft tissue or bleeding.

Moreover, the available anatomies were primarily long bones, such as the tibia, femur, and humerus. Contrarily, the pelvis has been identified in literature as a particularly challenging anatomical region for surgical resections due to its complex orientation and bone positioning [23, 24]. Given this, the integration of 3D-assisted technologies in pelvic tumor resections represents an area of significant interest. Compared to other studies cited in this article, the sample size of this study is smaller. This is partially a result of not repeating osteotomies to achieve independence. Furthermore, we tried not use resections that were similar to each other (e.g., multiple transverse tibial cuts, see Fig. 3) to maintain independence between cuts. This study scope only included osteosarcoma, but its results are likely to apply to other extremity bone resections, for example, for other malignant tumors like Ewing sarcoma.

## Conclusions

This study acts as a non-clinical proof of concept that the adoption of patient-specific instrumentation or mixed reality techniques for osteosarcoma resection might enable narrower margins pending in-vivo validation, potentially enabling bone and joint preservation and restoration, while decreasing resection failure rates.

## Supplementary Information

Supplementary Material 1.

## Data Availability

Data is provided in aggregated form within the manuscript. The dataset used and analyzed during the current study are available from the corresponding author on reasonable request.
