# Supplementary material for: Assessing the accuracy of 3D assistive technologies for surgical guidance of osteosarcoma resections: a comparative laboratory study of mixed reality, patient-specific instruments and freehand approaches
Source: Arthroplasty. 2026 Feb 5;8:9. doi: 10.1186/s42836-026-00369-8 (PMC12874771; doi:10.1186/s42836-026-00369-8)
Supplement: Supplementary file 1 — Supplementary Material 1. [file 42836_2026_369_MOESM1_ESM.pdf]

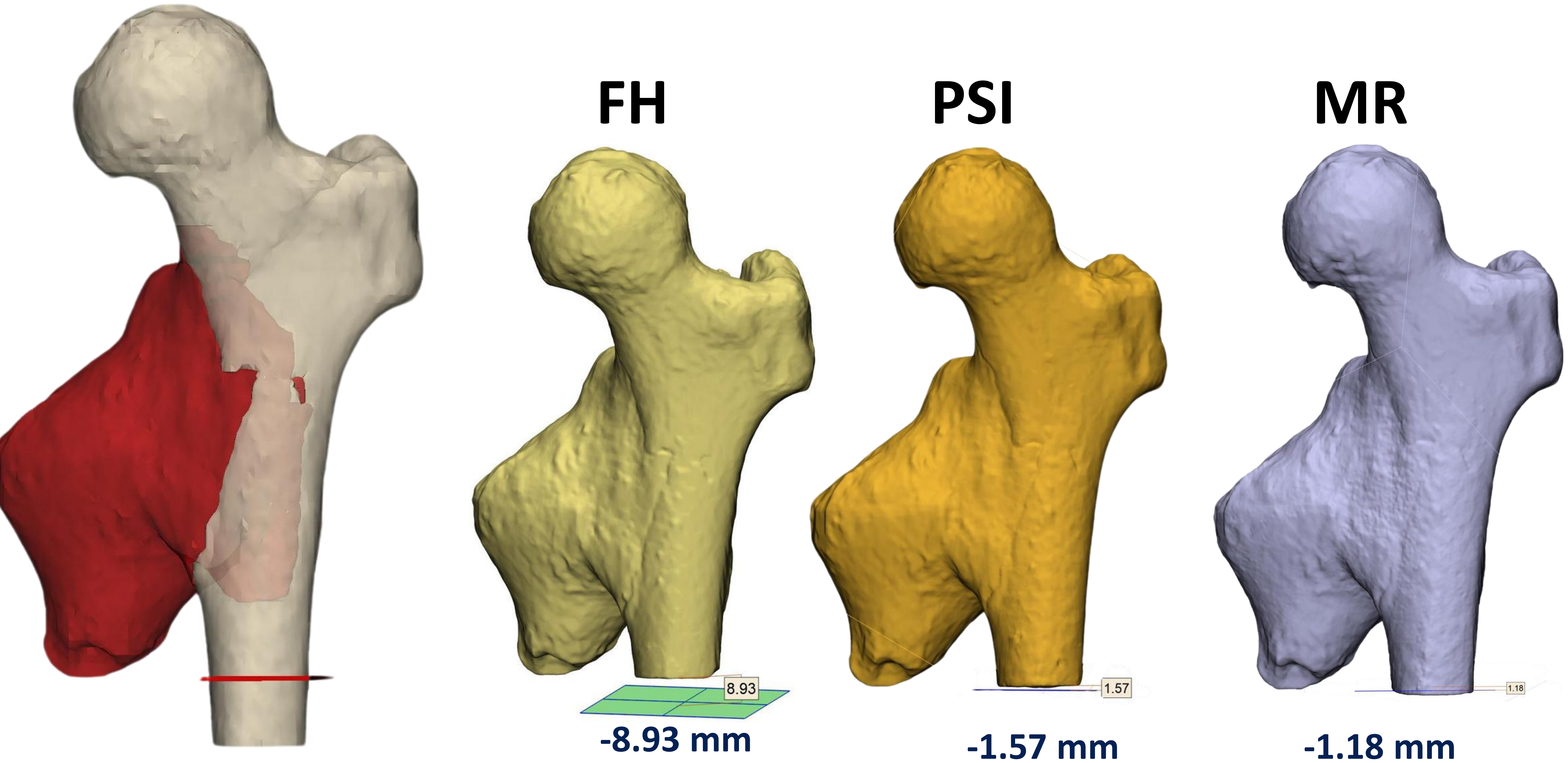

Supplemental Figure 1: *Comparison of proximal femur cuts*. The cuts were planned on the 3D model acquired from CT. After executing the resections, the models were 3D scanned and overlayed with the plan. Resulting largest distances between the model to the planned planes were measured.

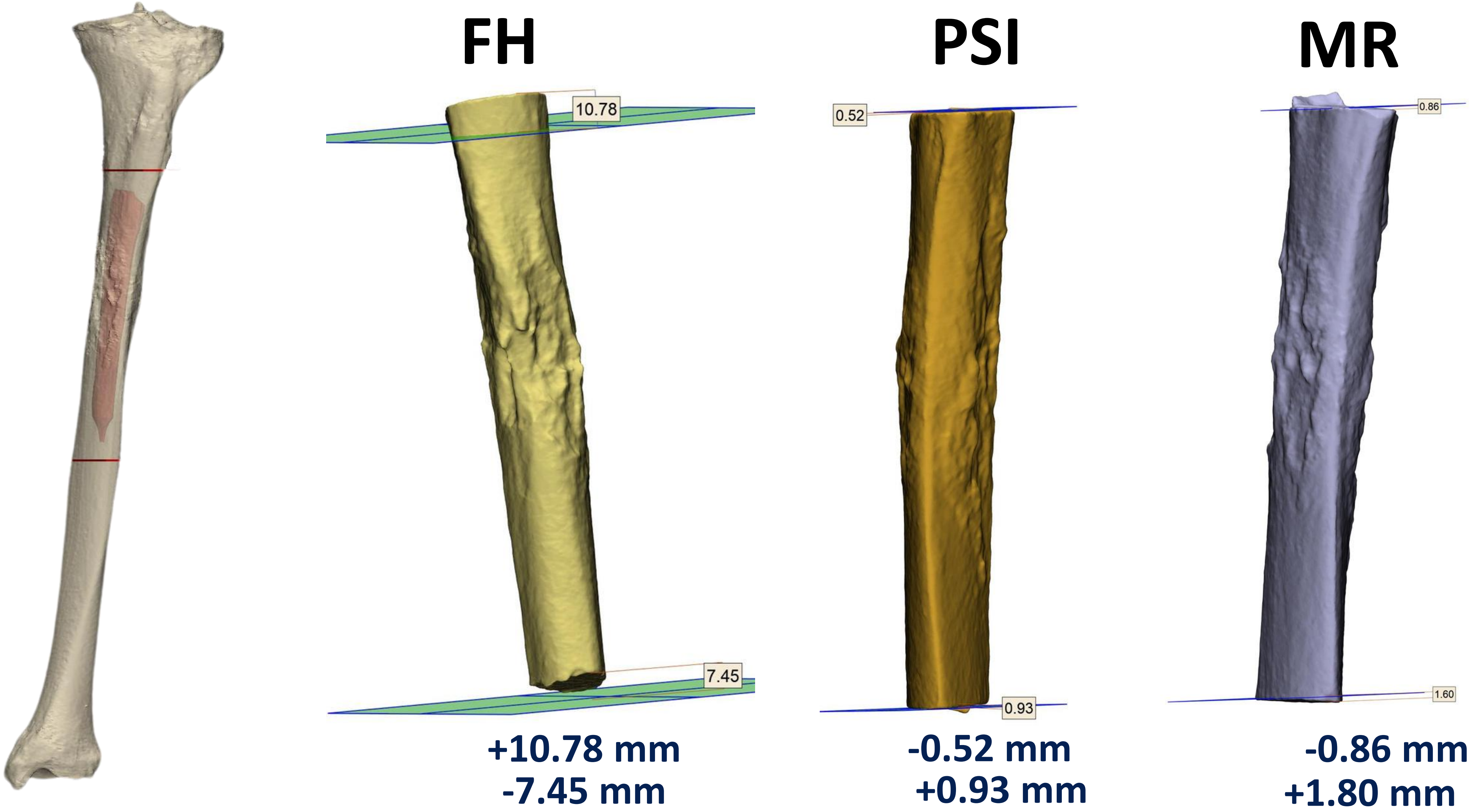

Supplemental Figure 2: *Comparison of proximal femur cuts*. The cuts were planned on the 3D model acquired from CT. After executing the resections, the models were 3D scanned and overlayed with the plan. Resulting largest distances between the model to the planned planes were measured.

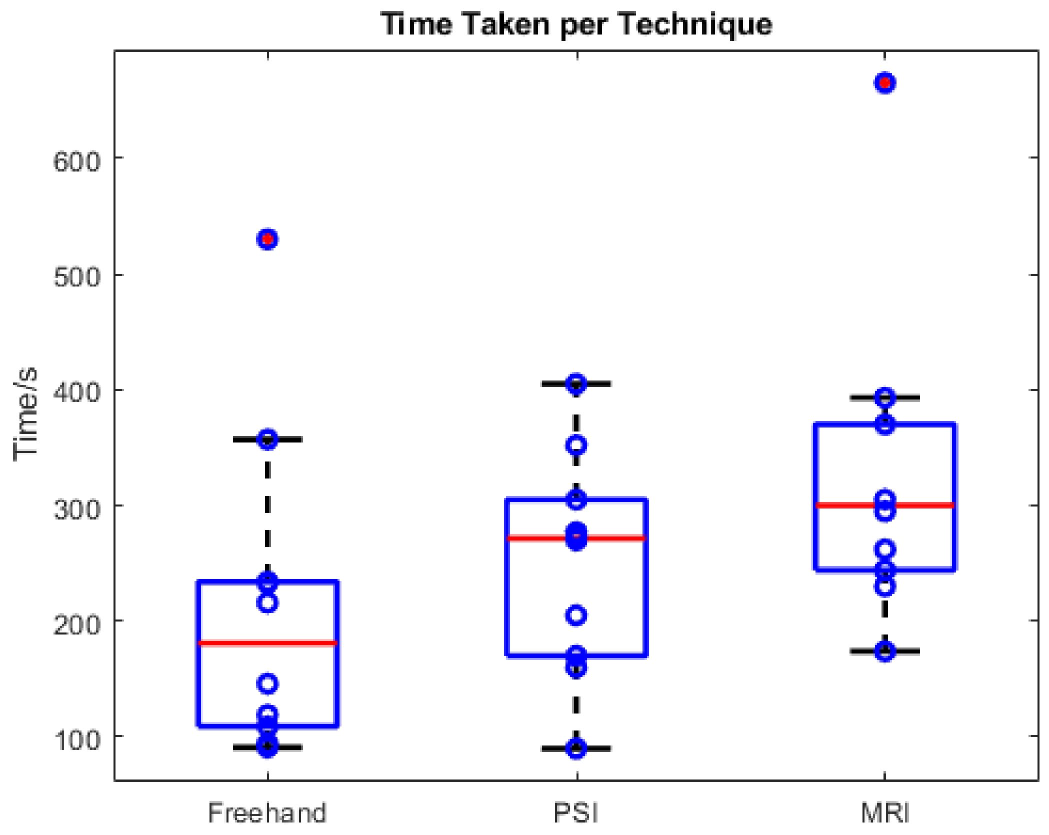

Supplemental Figure 3: *Comparison between time taken for techniques*. The time the operator took was measured from starting at the 3D printed model (i.e., registration of the MR device or putting on the PSI was included) until they decided they are finished with the model. Some models had multiple cuts and took therefore longer.
